# Phosphorylation Alters the Interaction of the Arabidopsis Phosphotransfer Protein AHP1 with Its Sensor Kinase ETR1

**DOI:** 10.1371/journal.pone.0024173

**Published:** 2011-09-02

**Authors:** Benjamin Scharein, Georg Groth

**Affiliations:** Biochemical Plant Physiology, Heinrich-Heine Universität, Düsseldorf, Germany; Ohio State University, United States of America

## Abstract

The ethylene receptor ethylene response 1 (ETR1) and the *Arabidopsis* histidine-containing phosphotransfer protein 1 (AHP1) form a tight complex *in vitro*. According to our current model ETR1 and AHP1 together with a response regulator form a phosphorelay system controlling the gene expression response to the plant hormone ethylene, similar to the two-component signaling in bacteria. The model implies that ETR1 functions as a sensor kinase and is autophosphorylated in the absence of ethylene. The phosphoryl group is then transferred onto a histidine at the canonical phosphorylation site in AHP1. For phosphoryl group transfer both binding partners need to form a tight complex. After ethylene binding the receptor is switched to the non-phosphorylated state. This switch is accompanied by a conformational change that decreases the affinity to the phosphorylated AHP1. To test this model we used fluorescence polarization and examined how the phosphorylation status of the proteins affects formation of the suggested ETR1−AHP1 signaling complex. We have employed various mutants of ETR1 and AHP1 mimicking permanent phosphorylation or preventing phosphorylation, respectively. Our results show that phosphorylation plays an important role in complex formation as affinity is dramatically reduced when the signaling partners are either both in their non-phosphorylated form or both in their phosphorylated form. On the other hand, affinity is greatly enhanced when either protein is in the phosphorylated state and the corresponding partner in its non-phosphorylated form. Our results indicate that interaction of ETR1 and AHP1 requires that ETR1 is a dimer, as in its functional state as receptor *in planta*.

## Introduction

The plant hormone ethylene influences many processes in plant growth and development. Reverse genetics has identified a family of membrane-bound sensor kinases (ETR1, ERS1, ETR2, ERS2, EIN4) as receptors of the gaseous hormone [1]–[4]. Sequence homology of these receptors with sensor kinases in typical bacterial two-component signaling systems suggest that a histidine-aspartate phosphoryl-group transfer could be also employed in ethylene signaling [5]. In ETR1 representing the prototype of the ethylene receptor family, signal perception by the amino-terminal domain of the sensor kinase is proposed to control autophosphorylation of a conserved histidine in its catalytic transmitter domain. Then this phosphoryl group is supposed to be transferred to a conserved aspartate in the carboxyl-terminal receiver domain of the receptor. This is in contrast to typical bacterial two component systems which are characterized by a single histidine to aspartate phosphotransfer step between two individual proteins - a sensor kinase and a response regulator [6], [7]. In ethylene signaling an additional phosphotransfer step is required to transfer the signal from the receiver domain of the membrane-localized sensor kinase ETR1 to the nuclear-localized response regulator proteins that control the cellular response to the plant hormone [5], [8], [9]. Connection between these two signaling modules is thought to be provided by histidine-containing phosphotransfer (HPt) proteins which transfer a phosphoryl group from the receiver domain of the receptor to the response regulator [10]. This type of multistep phosphorelay mechanism has been demonstrated unequivocally for signal transduction of the plant hormone cytokinin by the histidine kinases AHK2, AHK3, AHK4(CRE1) [11]–[14] and is also discussed for osmoregulation by the sensor kinase AHK1 [15].

The idea that a phosphorelay system is involved in ethylene signaling is further supported by yeast two-hybrid analyses which showed that the HPt proteins AHP1 and AHP3 can interact with the membrane extrinsic part (amino acids 321–721) of the ethylene receptor ETR1 but also with the A-type response regulator protein ARR4 [16]. Involvement of the B-type response regulator ARR2 in ethylene signaling was demonstrated by analyses of loss-of-function and over-expression lines as well as functional assays in protoplasts [17]. Furthermore, direct interaction of full-length ETR1 (amino acids 1–738) with the HPt protein AHP1 was recently demonstrated by fluorescence polarization studies providing quantitative analysis on the complex stability [18]. Although these experiments clearly emphasize that the receiver domain of the sensor kinase ETR1 forms a tight complex with the soluble cytoplasmic AHP1, they cannot answer whether phosphoryl-group transfer according to two-component signaling plays a functional role in ethylene signaling.

To address this question, we have examined how the phosphorylation status of both, the sensor kinase and the HPt protein, affects formation of the ETR1−AHP1 signaling complex. Moreover, we have analyzed how binding of an ethylene agonist which is known to reduce phosphorylation activity of the ETR1 sensor kinase [19] controls complex formation. Our data presented here show that phosphorylation indeed plays an essential role in complex formation as affinity is dramatically reduced when both signaling partner are in their non-phosphorylated or phosphorylated form, respectively. On the other hand complex formation is promoted when either AHP1 or ETR1 are in their phosphorylated form and the corresponding binding partner is set to its non-phosphorylated status. Analysis of the effective molar volume of the AHP1−ETR1 signaling complex in our sensitive fluorescence assay revealed that complex formation involves a dimer of the sensor kinase as is the functional state of the ETR1 receptor *in planta* [20]. Consequently, detailed analysis of steady state fluorescence polarization data from recombinant proteins in a controlled *in vitro* system provide an excellent approach to understand some of the signaling processes involved in ethylene signaling in a quantitative context.

## Results

### Analysis of the ETR1−AHP1 signaling complex in response to the phosphorylation status of ETR1 and AHP1

**Figure 1:**
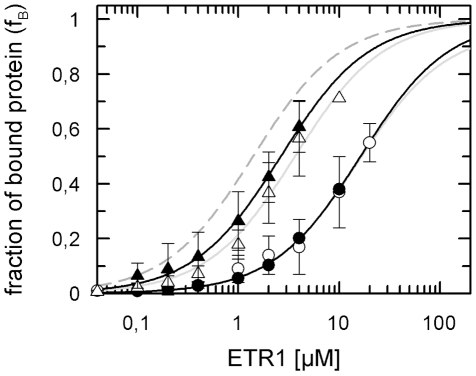
Binding curves of phosphorylation mutants of the sensor kinase ETR1 and the histidine transfer protein AHP1. Purified recombinant ETR1(D659A) or ETR1(D659E) at concentrations from 0.04 µM to 20 µM were added to 8 nM fluorescent AHP1-GFP(S65T) in a medium consisting of 50 mM Tris-HCl pH 7.5, 100 mM potassium chloride, 0.1% (w/v) β-D-dodecylmaltoside and 0.002% (w/v) phenylmethylsulfonyl fluoride. For the fluorescent AHP1 either non-phosphorylatable mutant AHP1(H79A)-GFP(S65T) or mutant AHP1(H79E)-GFP(S65T) mimicking permanent phosphorylation were used. Dissociation constants of the mutant ETR1−AHP1 complexes were calculated from the binding curves by Equation 2. ETR1(D659A) and AHP1(H79E)-GFP(S65T) show a K_d_ of 3.6 µM (Δ,——), ETR1(D659E) and AHP1(H79A)-GFP(S65T) a K_d_ of 2.6 µM (▴,——), ETR1(D659E) and AHP1(H79E)-GFP(S65T) a K_d_ of 16.3 µM (•,——) and ETR1(D659A) and AHP1(H79A)-GFP(S65T) a K_d_ of 15.4 µM (○,——). The dashed grey curve (—) corresponds to the binding characteristics of wild type ETR1 and wild type AHP1 [18].

In previous studies we have established a sensitive fluorescence polarization assay to validate and to quantify the interaction of the HPt protein AHP1 with the ethylene receptor ETR1 [18]. To gain further insight into this interaction we have analyzed the impact of phosphorylation on complex formation. Substitution of the canonical phosphorylation sites in ETR1 or AHP1 is supposed to interrupt phosphoryl group transfer in the putative ETR1−AHP1 signaling complex providing that phosphorylation plays a functional role as in typical bacterial two-component phosphorelay modules [21]. In order to mimic permanent phosphorylation in the receiver domain of the receptor or in the HPt protein we have substituted the putative canonical phosphorylation sites aspartate-659 in ETR1 and histidine-79 in AHP1 by glutamate (ETR1-D659E, AHP1-H79E), respectively. Due to its negative charge and comparable surface volume glutamate acts as a suitable structural substitute for the phospho-aspartate [22] and phospho-histidine residues [23] that are formed in AHP1 and ETR1 upon phosphorylation (see Supplement Figure S1). Substitution by alanine represents the non-phosphorylated state of the proteins (ETR1-D659A, AHP1-H79A). Interaction of the mutant proteins was analyzed according to the protocol described for the wild type proteins [18]. In short, purified recombinant ETR1 (up to 20 µM final concentration) was mixed to 8 nM of a fusion of AHP1 to a green fluorescent protein (AHP1-GFP(S65T)) in a medium containing 50 mM Tris(hydroxymethyl)-aminomethan (Tris-HCl) pH 7.5, 0.1 M potassium chloride, 0.1% (w/v) β-D-dodecylmaltoside and 0.002% (w/v) phenylmethylsulfonyl fluoride (PMSF). Formation of the complex was deduced from an increase in fluorescence polarization that is caused by the slower rotation and the associated increased rotational correlation time of the ETR1−AHP1-GFP(S65T) complex compared to free AHP1-GFP(S65T). Figure 1 shows binding curves obtained from titration of the non-phosphorylatable ETR1-D659A mutant with the non-phosphorylatable AHP1-H79A mutant and with the AHP1-H79E mutant which corresponds to a permanently phosphorylated form of the HPt protein, respectively. Binding curves of ETR1-D659E with mutant AHP1-H79E which both represent their permanently phosphorylated forms as well as with mutant AHP1-H79A where phosphorylation at the canonical phosphorylation site is abolished are also given in Figure 1. Compared to the wild type proteins which show a K_d_ = 1.4 µM [18], the dissociation constants obtained from these binding curves show a 10-fold increase (K_d_ = 16.3 µM for ETR1-D659E−AHP1-H79E and K_d_ = 15.4 µM for ETR1-D659A−AHP1-H79A). These results show that the interactions in the complex are much weaker when ETR1 and AHP1 are either both in their phosphorylated or both in their non-phosphorylated form. In contrast, dissociation constants obtained for ETR1−AHP1 when one of the binding partner mimics the phosphorylated form and the other one corresponds to the non-phosphorylated form, are almost the same as observed for the wild type (K_d_ = 2.6 µM for ETR1-D659E−AHP1-H79A and K_d_ = 3.6 µM for ETR1-D659A−AHP1-H79E). Taken together, these results clearly demonstrate that the interaction between AHP1 and the ethylene receptor ETR1 is modulated by the phosphorylation at the canonical phosphorylation sites of both proteins.

### Analysis of the ETR1−AHP1 signaling complex in response to ethylene agonists

**Figure 2:**
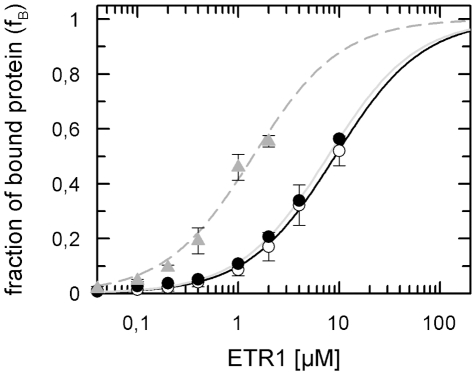
Binding curves of the sensor kinase ETR1 and histidine transfer protein AHP1. Measurements were done in the presence of 100 µM ethephon (•) or 100 µM potassium cyanide (○). In a control experiment (▴) the cofactor copper essential for ethylene binding was omitted. The dashed grey curve (—) represents a fit to these data according to Equation 2 corresponding to a dissociation constant of 1.4 µM [18]. Binding curves fitted to the experimental data obtained in the presence of ethephon (——) or in the presence of cyanide (——) correspond to dissociation constants of 7.7 µM and 9 µM, respectively.

Fluorescence polarization measurements were also employed to address the question whether and to what extent the ETR1−AHP1 interaction is affected by the plant hormone ethylene - the natural trigger of the signaling cascade. As ethylene is a highly volatile compound potassium cyanide, a well-documented ethylene agonist that mimics ethylene action and responses *in planta* [24], [25], and ethephon, a chemical that disintegrates into ethylene, phosphate and chloride, were applied in these studies. Wild type ETR1 was pre-incubated with 0.1 mM copper chloride which is an essential cofactor for ethylene binding [26], before the receptor was added to a buffer containing wild type AHP1-GFP, 50 mM Tris-HCl pH 7.5, 100 mM potassium chloride, 0.1% (w/v) β-D-dodecylmaltoside and 0.002% PMSF. Figure 2 illustrates the effect of ethephon and cyanide on the ETR1−AHP1 complex formation. Dissociation constants found in the presence of these ethylene agonists are increased by a factor of 5–6 compared to the dissociation constant obtained in the absence of a ligand (K_d_ = 9.0 µM for ETR1−AHP1 with cyanide and K_d_ = 7.7 µM for ETR1−AHP1 with ethylene). Binding curves obtained in the presence of cyanide or ethephon, but in the absence of the essential copper cofactor correspond to those of wild type ETR1 and AHP1 obtained in the absence of ethylene agonists. Hence, our results imply that not only the phosphorylation state of receptor and HPt protein but also the binding of the natural ligand, or an agonist, triggers conformational changes that affect the interaction in the ETR1−AHP1 complex.

### Determination of molecular volume and stoichiometry of the ETR1−AHP1 complex

**Figure 3:**
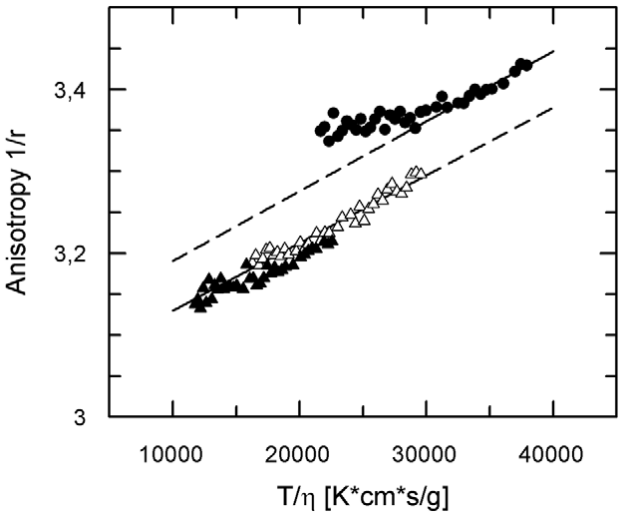
Perrin plot analysis of purified recombinant AHP1-GFP. Measurements were performed in a buffer containing 50 mM Tris-HCl pH 7.5, 100 mM potassium chloride, 0.1% (w/v) β-D-dodecylmaltoside and 0.002% (w/v) phenylmethylsulfonyl fluoride at temperatures between 10°C and 30°C and glycerol concentrations of 10% (w/v) (Δ) and 20% (w/v) (▴). Closed circles (•) correspond to measurements at 0% (w/v) glycerol. At these conditions an increase in the molecular volume of the GFP-fusion protein is observed at temperatures below 17°C probably due to protein aggregation.

In addition to detailed information on complex formation and complex stability steady-state fluorescence polarizations studies can also provide information on the molecular volume and the stoichiometry of a protein complex. Measurements of fluorescence polarization of 20 nM AHP1-GFP at different temperatures and/or viscosities of the medium are summarized in Figure 3 which shows a Perrin plot, a plot of the reciprocal fluorescence anisotropy versus the temperature/viscosity ratio of the medium. From the slope and the intercept of the plot the molar volume and thereby the apparent molecular weight of the purified recombinant AHP1-GFP (M_w_ monomer 45 kDa) was calculated according to equation (4). The calculated molecular weight of 90 kDa suggests that the protein forms a dimer at these conditions. Due to the unfavorable ratio of GFP fluorescence lifetime and rotational correlation time of complexes above 100 kDa we changed the fluorophor and employed 5-(dimethylamino)naphthalene-1-sulfonyl chloride (dansyl chloride) as fluorescent reporter for the analysis of the apparent molecular weight of ETR1 and for the stoichiometry of the ETR1−AHP1 complex. Proteins were dansylated as described in Material and Methods and analyzed at different temperatures and viscosities according to the protocol described for AHP1-GFP.

**Figure 4:**
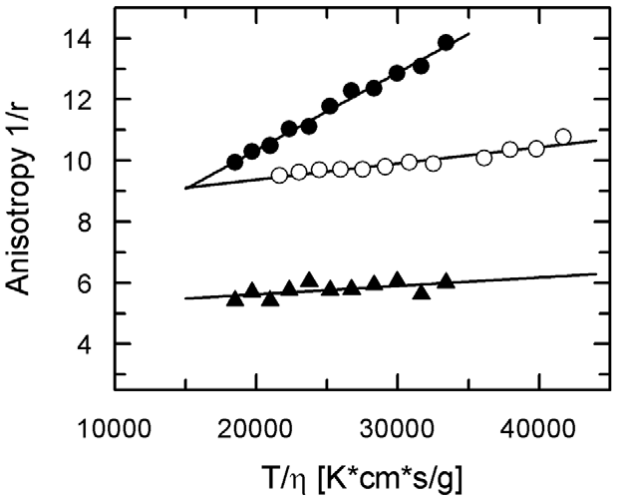
Perrin plot analysis of recombinant ETR1, AHP1 and ETR1−AHP1 complex. Purified proteins were dansylated and fluorescence anisotropy was recorded in 0.2 M potassium phosphate pH 8.0, 0.05% (w/v) β-D-dodecylmaltoside and 10 mM β-cyclodextrin at temperatures between 5°C and 25°C. Curves correspond to data obtained with 0.2 µM dansylated AHP1 (•), 0.2 µM dansylated ETR1 (○) and 2 µM ETR1 together with 0.2 µM dansylated AHP1 (▴).

From the Perrin plot which is shown in Figure 4 an apparent molecular weight of 42 kDa was obtained for the dansylated AHP1 (M_w_ monomer 18 kDa), while the molecular size of dansylated ETR1 (M_w_ monomer 85 kDa and M_w_ dodecylmaltoside micelle 72 kDa [27]) was estimated to 280 kDa. Both data which were confirmed in three independent measurements indicate that both recombinant proteins, AHP1 and also ETR1 form homodimers at the conditions applied in the fluorescence polarization assay. Note that for AHP1, dimerization was already observed with the GFP-fusion. Analysis of the ETR1−AHP complex was done in the same medium as for the individual proteins at 0.2 µM AHP1 and 2 µM ETR1. From the Perrin plot an apparent molecular weight of approximately 350 kDa was obtained for the ETR1−AHP1 complex suggesting that the signaling complex contains a homodimer of each of the receptor and of the HPt protein.

## Discussion

In the present work we have studied intermolecular interactions of a protein complex involved in ethylene signaling by steady state fluorescence polarization. We have demonstrated that phosphorylation at a canonical phosphorylation site of the HPt protein AHP1 reduces its affinity for a mutant of the membrane sensor ETR1 that mimics the phosphorylated form of the kinase by at least 10-fold. The same clear reduction in affinity was observed when non-phosphorylatable mutants of both binding partner were used. The apparent dissociation constant for the interaction of either both phosphorylated or both non-phosphorylated proteins was approximately 15 µM which corresponds to the low dissociation constant of 17 µM obtained for the typical bacterial two component system CheA−CheW [28]. When the kinase or the transfer protein, respectively, mimics its phosphorylated form (ETR1-D659E or AHP1-H79E) and the corresponding binding partner its non-phosphorylatable form (ETR1-D659A or AHP1-H79A), the interaction is tight. These complexes show dissociation constants of 2–3 µM which are similar to those observed for the recombinant wild type proteins. This finding suggests that both proteins are partly phosphorylated when purified from the bacterial host. Alternatively, wild type ETR1 that is purified at denaturing conditions and refolded to its active conformation after purification [19] is present in its non-phosphorylated state, while the natively purified AHP1 is in its phosphorylated form. Nevertheless, a charged residue, i. e. either the phosphorelay phosphoryl group or a genetically placed negatively charged glutamate which has been shown in previous studies to efficiently mimic the phosphorylated state of a protein kinase [29], seem to be necessary for tight interaction of the sensor kinase with its HPt protein. The idea that phosphorylation at the canonical sites affects the stability of the sensor kinase–HPt protein complex is further supported by yeast two hybrid studies on the hybrid-type histidine kinase ATHK1 [16]. When the putative phosphorylation site aspartate-1074 in the receiver domain of ATHK1 was substituted by glutamate and expressed in yeast, the mutant showed no interaction with AHP1 - an HPt protein that was previously shown to interact with the wild type receiver domain of ATHK1 [16]. This result indicates that the phosphorylation state of the osmosensor is essential for the binding of AHP1. Yeast two-hybrid analysis did not resolve the phosphorylation state of the canonical phosphorylation site since it could not be discriminated whether D1074 is phosphorylated by crosstalk with other two component systems when expressed in yeast. Hence, it seems possible that only the non-phosphorylated receiver domains of ATHK1 can interact with HPt domains, and that a phosphorylated receiver domain shows only weak interaction which is not resolved by yeast two hybrid analysis. The fact that phosphorylation in the receiver domain has a negative effect on the interaction with the non-phosphorylated HPt protein seems to contradict the results obtained in this study. However, this apparent paradox might be resolved by the fact that in contrast to the osmosensor ATHK1, ethylene receptors are negative regulators which are in the absence of the plant hormone in their phosphorylated state [19].

In contrast to yeast two hybrid studies the assay presented in this study provides not only a quantitative read-out of the interaction, but also the opportunity to test the effect of the natural trigger ethylene or its agonists on the interaction of sensor kinase and HPt protein. Measurements obtained in the presence of cyanide or ethephon - a chemical releasing ethylene *in situ* - indicate that the affinity of both binding partner in the ETR1−AHP1 complex is reduced by a factor of 5. These results suggest that a conformational change in the receiver domain triggered by the binding of the plant hormone is necessary in addition to the electrostatic requirements in the receiver domain to regulate tight interaction with the HPt protein. Controls obtained in the absence of essential copper cofactor where binding of the plant hormone is prevented showed no effect of cyanide or ethephon on the ETR1−AHP1 interaction and support this analysis.

In addition to quantitative analysis and evaluation of the effect of ethylene agonists on the interaction of the sensor kinase ETR1 and the HPt protein AHP1 steady state fluorescence polarization studies also provide information on the stoichiometry of the signaling complex. Analysis of the polarization data according to Perrin (see Fig. 4) revealed that recombinant ETR1 and AHP1 are forming a tetrameric complex consisting of two homodimers at the conditions applied in the *in vitro* assay. Taking into account the theoretical molecular weight for AHP1 (18.3 kDa), AHP1-GFP (45.6 kDa) and ETR1 (84.9 kDa) the data determined from the individual proteins and the ETR1−AHP1 complex account for an AHP1 dimer (41±5 kDa for dansylated AHP1 and 89.8±1.4 kDa for AHP1-GFP), a detergent-solubilized ETR1-dimer (282±21 kDa) considering an average dodecylmaltoside micelle size of 72 kDa [27], and for an ETR1−AHP1 dimer (347±30 kDa) in a dodecylmaltoside micelle. Studies on ethylene receptors ETR1 and ERS1 of *Arabidopsis* indicate that they form disulfide-linked homodimers *in planta* [20], [30]. Moreover, membrane recruitment studies demonstrated that all five members of the *Arabidopsis* ethylene receptor family form homomeric and heteromeric protein complexes at the ER in living plant cells [31]. Similarly, HPt proteins might also form dimers *in vivo* as indicated by the dimerization of the HPt protein Spo0B from *Bacillus subtilis* [32]. Other HPt proteins such as the phosphorelay protein YPD1 [33], the ArcB HPt domain [34], and the CheA P1 domain [35] are probably monomers in their functional form.

**Figure 5:**
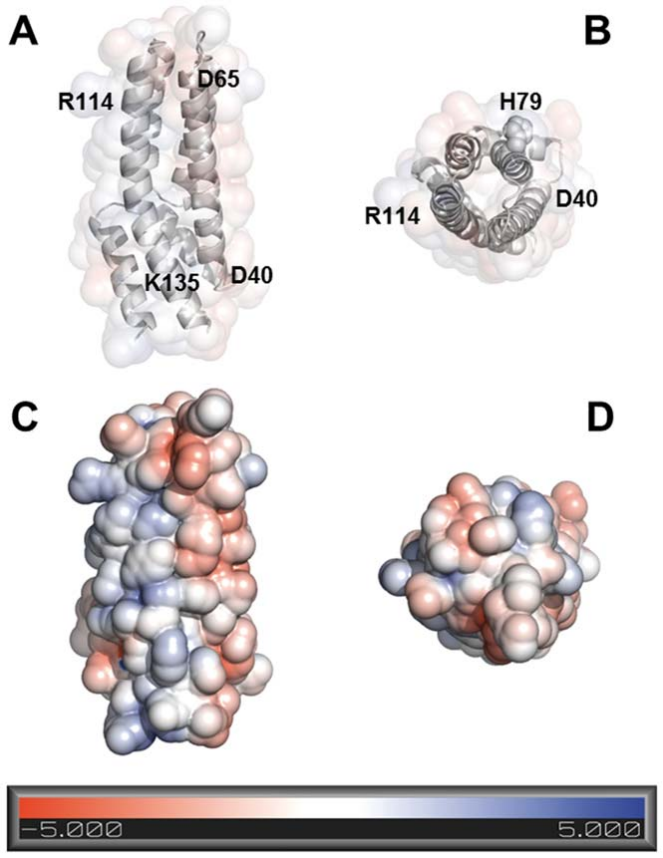
Electrostatic surface potential of the histidine phospho-transfer protein AHP1. A structural model of AHP1 was built using *MODELLER* [44] interfaced by *EASYMODELLER* [45] with default options using the 3D structure of the histidine-containing phosphotransfer protein OsHP1, from rice (PDB code 1YVI). In this method the 3D-structure is generated in such a way that a set of spatial and empirically determined restraints are optimally satisfied. Electrostatic potentials for this model were calculated using *PDB2PQR* [46] and *APBS* [47]. Images were generated with *PyMOL* [48]. Solvent-accessible surface is colored according to the electrostatic potential [−5 kT/e red, +5 kT/e blue]. (A) and (C) side view, (B) and (D) top view. Catalytic residue H79 and residues defining the negative (residues D40-D65) and positive electrostatic surface potential (residues R114-K135) are labelled.

A homology model of AHP1 built on the crystal structure of the Hpt protein OsHP1 from rice (PDB code 1YVI) which is shown in Figure 5 further supports our experimental data that AHP1 forms homodimers in solution. The Poisson-Boltzmann electrostatic potential which is displayed on the protein surface clearly indicates a negatively charged (Helix 3, residues D40-D65) and a positively charged arm (Helix 6, residues R114-K135). Formation of the AHP1 homodimer might occur via these two complementary electrostatic surfaces when two monomers interact in a back-to-back orientation (aHelix3-bHelix6 and aHelix6-bHelix3). The catalytic histidine-79 in both monomers are well accessible in this putative complex as they are facing the opposite site of the interacting surfaces provided by Helices 3 and 6 in both monomers.

Taken together, the *in vitro* fluorescence assay presented in this study employs the proteins in the same functional state as observed *in planta* and in bacteria.

**Figure 6:**
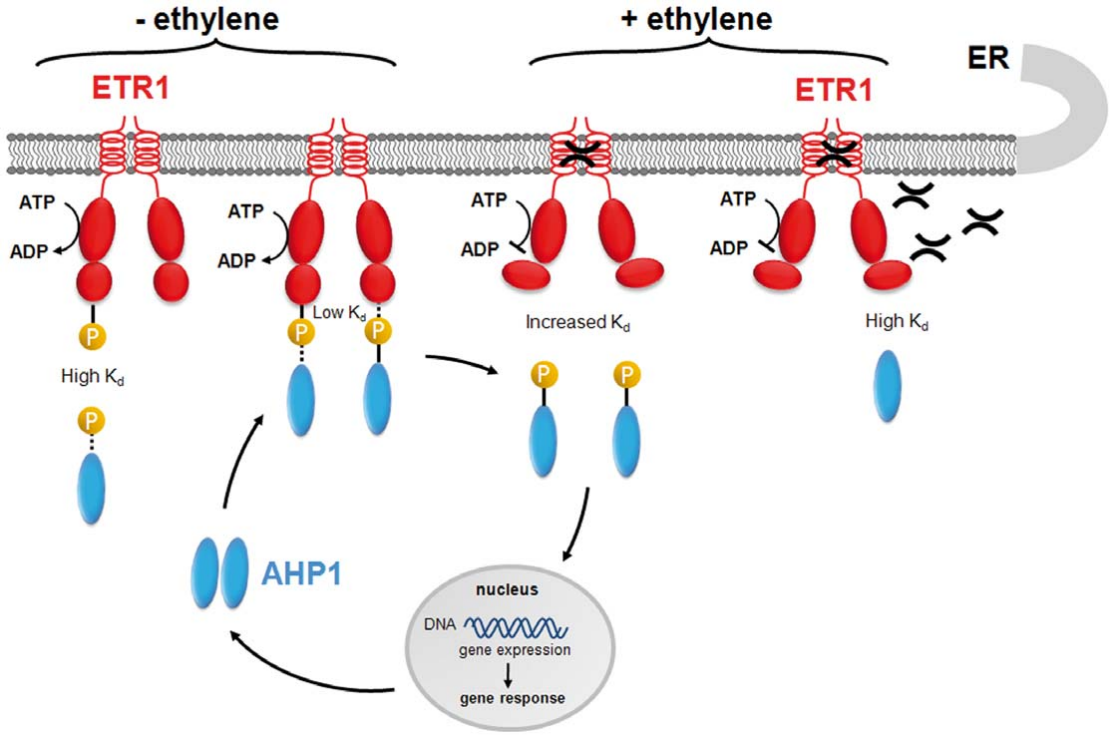
Schematic model of ETR1−AHP1 signaling. In the absence of ethylene autophosphorylation of the sensor kinase ETR1 ensures low affinity of the receptor to phosphorylated HPt proteins. On the other hand, non-phosphorylated AHP1 binds to the phosphorylated ETR1 receptor with high affinity enabling phosphoryl group transfer to the HPt protein. After ethylene binding the receptor is switched to the non-phosphorylated state. This switch is accompanied by a conformational change that decreases the affinity to the phosphorylated AHP1. The transfer protein, AHP1-P is released and can move to the nucleus for further signal transfer onto response regulator proteins causing gene response. Binding of non-phosphorylated AHP1 to the activated form of the receptor is prevented by the conformational change caused in the receiver domain of the receptor by the binding of the plant hormone.

Based on the results presented in this study, we propose that the phosphorylation state has an important role on ethylene signal transduction and suggest the schematic model shown in Figure 6 to describe the effect of the phosphorylation state of the receptor kinase on the interaction with the HPt protein. At standard conditions ETR1 is phosphorylated by its intrinsic autokinase activity and non-phosphorylated AHP1 binds with high affinity to the phosphorylated receiver domain of the sensor kinase. In turn the phosphoryl group is transferred to the canonical histidine in AHP1. The complex keeps the tight binding preventing signal transfer of the phosphoryl group to response regulator proteins in the nucleus. At this condition the signaling cascade is shut-off. Binding of the natural trigger of the sensor kinase - the plant hormone ethylene - is likely to cause a conformational shift in the receiver domain as indicated by the increased K_d_ of the ETR1−AHP1 complex observed in the presence of cyanide or ethephon. Decrease in the affinity of both binding partners promotes dissociation of the phosphorylated HPt protein from the receptor and further signal transfer to nuclear response regulators. Thereby AHP1 would regain its non-phosphorylated state which binds with high affinity only to the phosphorylated, but not to the non-phosphorylated ETR1 sensor kinase (see high K_d_ of alanine mutants of AHP1 and ETR1).

Interference of AHP1 that has been phosphorylated by other plant two-component based systems (e.g., cytokinin-receptors AHK2, AHK3, AHK4/CRE1/WOL [36]) resulting in an obstruction of these pathways is suspended by the low affinity of the phosphorylated HPt protein with the phosphorylated form of the ETR1 receptor (see high dissociation constant of the ETR1-D659E−AHP1-H79E complex) occurring in the absence of ethylene.

In summary our work emphasizes that individual steps in a signal transduction pathway have to be characterized separately in order to rationalize the mechanistic basis of phenotypes observed in molecular genetic studies.

## Materials and Methods

### Materials

Chemicals and reagents were purchased from Serva (Heidelberg, Germany), AppliChem (Darmstadt, Germany), Glycon (Luckenwalde, Germany), VWR International (Geldenaaksbaan, Belgium), BD (Le Pont de Claix, France) and Carl Roth (Karlsruhe, Germany) at analytical grade. Plasmids were derived from pET vectors from Merck/Novagen (Darmstadt, Germany). Oligonucleotides were from Sigma-Aldrich (Steinheim, Germany).

### Methods

#### Cloning, expression and purification

Site directed mutagenesis was carried out using the single-tube megaprimer PCR method as described in [37]. The plasmids pET16b_AtETR1 and pET21a_AtAHP1 used for mutagenesis were previously described [18]. Primers are listed in Table 1.

**Table 1:** Oligonucleotides used for mutagenesis.

Name	Sequence
5′ AHP1(H79A)	GAT CCC CAT GTT GCT CAA CTC AAA GGT AGC
5′ AHP1(H79E)	GAT CCC CAT GTT GAG CAA CTC AAA GGT AGC
T7 Promotor primer	TAA TAC GAC TCA CTA TAG GG
T7 Terminator primer	GCT AGT TAT TGC TCA GCG G
5′ ETR1(D659E)	GTC TTC ATG GAG GTG TGC ATG CCC GGG GTC GAA
5′ ETR1(D659A)	AAA GTG GTC TTC ATG GCC GTG TGC ATG

Plasmids encoding mutant AHP1 were transformed into *Escherichia coli* strain BL21 Gold (DE3) (Stratagene, La Jolla, USA). Proteins were expressed recombinantly and purified from the bacterial host by immobilized metal ion affinity chromatography under native conditions as described for wild type AHP1 [18]. Protein concentration was determined by the Bio-Rad protein assay (Bio-Rad, München, Germany) using bovine serum albumin as a standard. Plasmids coding for mutant ETR1 were transformed into *E. coli* strain C43 (DE3) [38]. Expression and purification was performed following the protocol described earlier [19]. Protein concentration of the purified ETR1 receptor was determined by the bicinchoninic acid assay (Perbio Science, Bonn, Germany) using bovine serum albumin as a standard.

#### Dansylation of ETR1 and AHP1

Purified proteins were concentrated in a buffer containing 0.2 M potassium phosphate, pH 8.0, and 0.05% (w/v) β-D-dodecylmaltoside. The protein solutions, 0.05 mg/ml (2,8 µM) AHP1 and 1 mg/ml (11,8 µM) ETR1, respectively, were incubated at a ratio of 1∶10 with 5% (v/v) of a freshly prepared 20-fold solution of dansyl chloride in dimethylformamide. The protein/dansyl chloride solution was incubated for 1 hour in the dark at room temperature. Final labeling ratio was determined spectrophotometically using a Beckman-Coulter DU800 spectrophotometer assuming a molar extinction coefficient of 4500 M^−1^ cm^−1^ at 340 nm [39]. Typical ratio of protein-dansyl labeling was 1∶4.

#### Fluorescence polarization assay

Fluorescence polarization measurements were performed in a LS55 Luminescence Spectrophotometer (Perkin Elmer, Rodgau-Jügesheim, Germany). For all experiments a SUPRASIL quartz 4×4 mm macro/semi-microcuvette (Perkin Elmer) with stirrer was used. Fluorescence polarization was calculated from parallel and perpendicular fluorescence intensities according to the following equation,
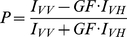
(1)where GF is the grating factor. Determination of the dissociation constant (K_d_) and fraction of bound protein (f_B_) was done by the equation,
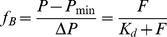
(2)where P is the measured polarization, *ΔP = P_max_−P_min_* is the difference between polarization before and after addition of the ETR1 receptor [40]. F corresponds to the concentration of free ETR1. The binding curve was obtained by plotting the normalized fluorescence polarization *f_B_* versus the ETR1 concentration. Dissociation constants were determined from this curve using the program GraFit (Erithacus Software, Horley, Surrey, U.K.) by a fit of the experimental data to a model assuming a single binding site in the interacting partners.

#### Analysis of molecular weight and stoichiometry of the AHP1−ETR1 complex

The molecular weight of AHP1, ETR1 and the AHP1−ETR1 complex was determined using the Perkin Elmer LS55 Spectrophotometer equipped with a Biokinetics Accessory, a PTP-1 Fluorescence Peltier System and a PCB1500 Water Peltier System (Perkin Elmer) by measuring anisotropy at different viscosities and temperatures of the medium. The following equation was used for calculating the anisotropy.
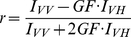
(3)The molecular weight M_w_ of the analyzed proteins was calculated according to
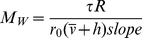
(4)where τ is the fluorescence lifetime, R the universal gas constant, 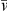 the specific volume and h the hydration of the protein. Typical values of 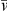 = 0.73 mL/g and h = 0.23 mL/g water per gram of protein were taken from [41]. Fluorescence lifetimes of 2.9 ns and 20 ns are listed for GFP [42] and dansyl chloride [43], respectively. Limiting anisotropy r_0_ and slope were obtained from a plot of the reciprocal fluorescence anisotropy versus the temperature/viscosity ratio of the medium according to Perrin.

An excitation wavelength of 489 nm and an emission wavelength of 510 nm were used for GFP. Slit width was set to 8.5 nm. For dansylated proteins the excitation wavelength was fixed to 333 nm and emission wavelength to 513 nm with a slit width of 5 nm.

## Supporting Information

Figure S1 Structural alignment of glutamate with phospho-aspartate (left panel) and phospho-histidine (right panel) from known protein structures. Structural information for phospho-aspartate were obtained from the response regulator protein from *Burkholderia pseudomallei* (pdb-code 3RQI) and from the receiver domain of the transcriptional regulatory protein FixJ from *Sinorhizobium meliloti* (pdb-code 1D5W). Coordinates for phospho-histidine were taken from Nucleoside Diphosphate Kinase from *Dictyostelium discoideum* (pdb-code 1NSP) and from *E. coli* Phosphoglycerate Mutase (pdb-code 1E58). Phospho-amino acids and glutamate were aligned in PyMol. Carbon atoms of glutamate are colored in red, while carbon atoms extracted from the pdb coordinates are shown in white. Nitrogen and oxygen are drawn in blue and red for all residues. Phosphor atoms are colored orange.
(TIF)
