# Supplementary figures and images for: Phosphorylation Alters the Interaction of the Arabidopsis Phosphotransfer Protein AHP1 with Its Sensor Kinase ETR1
Source: PLoS One. 2011 Sep 2;6(9):e24173. doi: 10.1371/journal.pone.0024173 (PMC3166298; doi:10.1371/journal.pone.0024173)

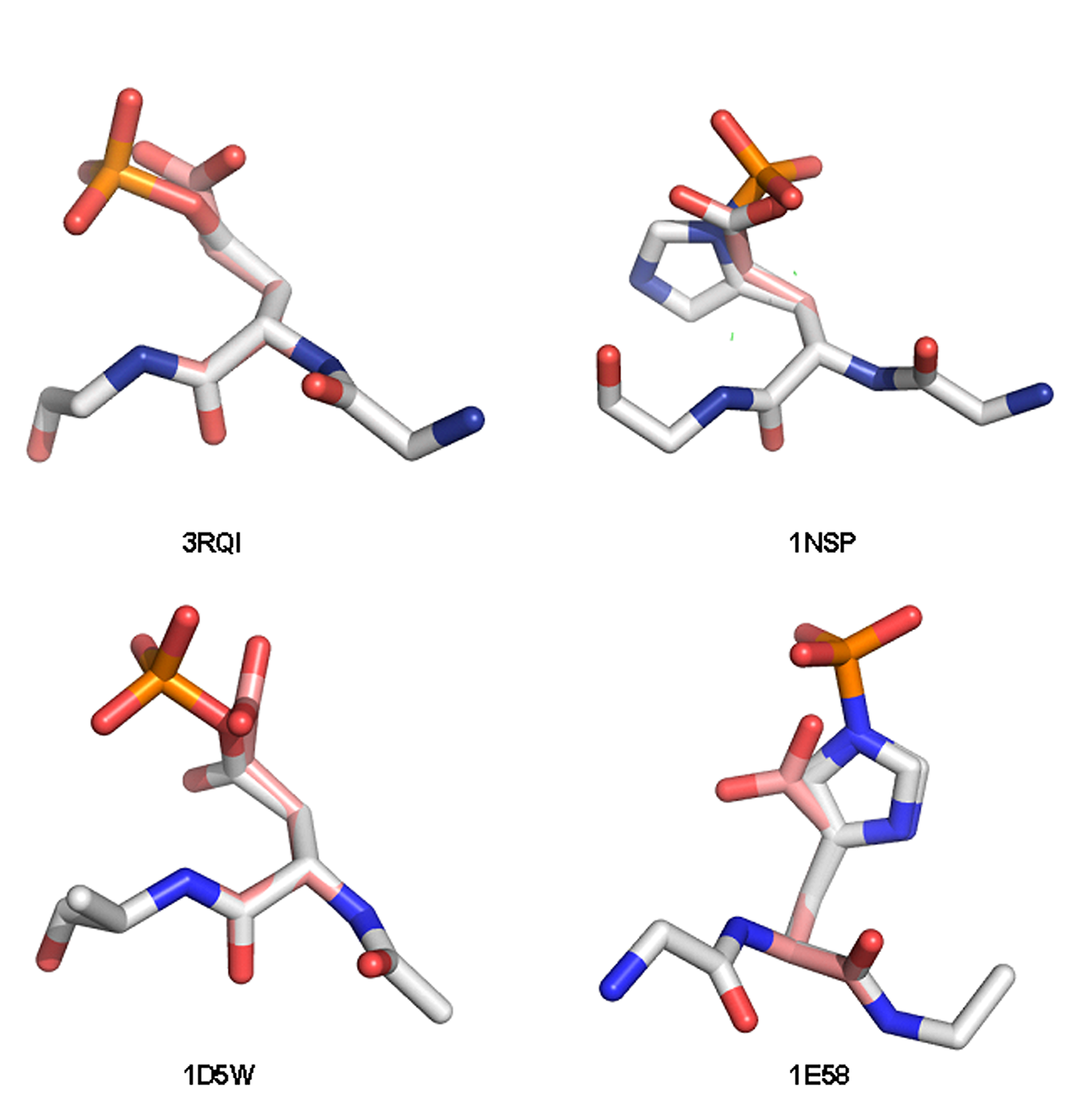

Supplement: Figure S1 — Structural alignment of glutamate with phospho-aspartate (left panel) and phospho-histidine (right panel) from known protein structures. Structural information for phospho-aspartate were obtained from the response regulator protein from Burkholderia pseudomallei (pdb-code 3RQI) and from the receiver domain of the transcriptional regulatory protein FixJ from Sinorhizobium meliloti (pdb-code 1D5W). Coordinates for phospho-histidine were taken from Nucleoside Diphosphate Kinase from Dictyostelium discoideum (pdb-code 1NSP) and from E. coli Phosphoglycerate Mutase (pdb-code 1E58). Phospho-amino acids and glutamate were aligned in PyMol. Carbon atoms of glutamate are colored in red, while carbon atoms extracted from the pdb coordinates are shown in white. Nitrogen and oxygen are drawn in blue and red for all residues. Phosphor atoms are colored orange. (TIF) [file pone.0024173.s001.tif]
